# Selections

**Published:** 1871-11

**Authors:** 


Electrolysis in the Treatment of Cancer.—At a meeting of the London Clinical Society, Dr. Lawson Tait related a case of encephaloid cancer of the femur treated by electrolysis. The patient had suffered excruciating pains for a long time, and for the relief of the same had tried morphia, chloral and other narcotics in vain. Ultimately he tried the effects of electrolysis on the tumor; he inserted (under chloroform) six needles into the tumor and applied the current for ten minutes, and three days after, repeated the operation for fifteen minutes. These operations gave the patient great immunity from suffering.

Dr. Althaus, before the same Society, stated that in fourteen cases treated by electrolysis, the tumors, whatever they might have been, disappeared, but that the two most decidedly successful cases were those of cancer of the breast.—*Lancet.*

Chloral Suppositories.—In the Lancet for October, G. W. Whidborne reports the following case, showing the good effects of hydrate of chloral in suppositories, when rejected by the mouth in every form:

"Mrs. Jane C——, aged forty, soon after the birth of her seventh child, suffered with severe and constant pains, which produced convulsions of a most fearful class. After all the usual remedies were made use of, without any permanent effect, I had recourse to the chloral hydrate suppositories: chloral hydrate, one drachm; hard soap, two scruples; honey, sufficient quantity. After using two of the suppositories, she had no more convulsions. She had four hours' good sleep, from which she awoke convalescent."

Chloral Hydrate and Cod-Liver Oil.—The *Gaz. Farm. Ital.* advocates the addition of chloral hydrate to cod-liver oil, which renders it much less nauseous, and prevents the night-sweats of the phthisical invalid, induces sound sleep, and creates appetite. It is prepared as follows: Ten grains of pure chloral hydrate crystals to one hundred and ninety grains of cod-liver oil, digested in a sand-bath. Dose, six tablespoonfuls daily.

Chloral in Asthmatic Bronchitis.—Dr. Caspar Morris ordered five grains of the chloral in one fluid-drachm of the syrup of lactucarium, to be repeated in two hours, if required. "The two doses afforded entire relief; and she has found great comfort since from a single dose taken at bed-time—a good night's rest being secured by it."—*Cincinnati Lancet and Observer.*

